# Management pathways and outcomes of children with hearing loss identified through newborn and infant hearing screening at a tertiary paediatric hospital in the Western Cape, South Africa

**DOI:** 10.4102/sajcd.v73i1.1187

**Published:** 2026-07-31

**Authors:** Silva Kuschke, Chéri van Zyl, Mmakgotso Mokete, Lebogang Ramma

**Affiliations:** 1 School of Medicine, Vanderbilt University, Nashville, Tennessee, United States of America; 2 Department of Health and Rehabilitation, Faculty of Health Sciences, University of Cape Town, Cape Town, South Africa; 3 Cayman Hearing Center, Grand Cayman, George Town, Cayman Islands

**Keywords:** newborn and infant hearing screening, management outcomes, amplification, early hearing detection and intervention (EHDI), South Africa

## Abstract

**Background:**

Early Hearing Detection and Intervention programmes support early identification and management of hearing loss. However, limited data exist on post-diagnostic pathways, amplification uptake, and continuity of care within public-sector paediatric services.

**Objectives:**

To describe management pathways and outcomes of children diagnosed with hearing loss following newborn and infant hearing screening (NIHS) at a tertiary paediatric hospital in the Western Cape.

**Method:**

A retrospective descriptive cohort study was conducted at the Red Cross War Memorial Children’s Hospital. Records of children under 6 years (August 2019 – August 2024) with documented NIHS results and confirmed hearing loss were reviewed using departmental databases and a self-developed data extraction tool. Data included demographics, risk factors, diagnostic outcomes, amplification, referrals, and follow-up attendance.

**Results:**

Of 7871 children seen, 511 (6.5%) had NIHS results. Eighty-three underwent diagnostic testing, of whom 72 (86.7%) were diagnosed with hearing loss. The mean age at diagnosis was 17.3 months. Infectious (32.5%) and neurological (28.9%) risk factors predominated. Mild (31.3%) and moderate (33.7%) conductive loss was most frequent. Only 15.3% received amplification, predominantly bilateral behind-the-ear devices. Most children (78.3%) were referred to additional services, primarily otolaryngology (81.5%). Follow-up attendance remained high (85.5%).

**Conclusion:**

High follow-up rates contrast with low uptake of amplification, likely reflecting conductive pathology and systemic constraints. Greater integration across audiology, otolaryngology, and early intervention services is needed.

**Contribution:**

This study provides contextually relevant evidence on post-diagnostic paediatric hearing care, including amplification uptake, referral patterns, and continuity to follow-up in the South African public sector.

## Introduction

Early Hearing Detection and Intervention (EHDI) frameworks emphasise early identification of hearing loss, and also timely and appropriate intervention to optimise speech, language, educational, and psychosocial outcomes (Joint Committee on Infant Hearing [JCIH], 2019). While newborn and infant hearing screening (NIHS) programmes aim to reduce the age of detection, diagnosis alone does not guarantee successful intervention or developmental benefit. International evidence increasingly recognises that successful EHDI programmes require timely amplification, medical management, family-centred interventions, and sustained follow-up throughout early childhood (JCIH, 2019; World Health Organization, 2021).

In lower-resourced countries, including South Africa, post-diagnostic management pathways are frequently fragmented. Barriers include resource constraints, limited access to multidisciplinary services, socioeconomic challenges, and geographical inequities (Kanji & Khoza-Shangase, 2016; McPherson, 2012). South African NIHS research has largely focused on screening coverage, referral rates, and age of diagnosis (Khoza-Shangase & Harbinson, 2015), with limited evidence describing rehabilitation trajectories, amplification uptake, and continuity of care. Despite increasing evidence regarding screening and diagnostic processes, comparatively little is known about post-diagnostic management trajectories within South African public-sector paediatric populations.

Existing South African EHDI literature has predominantly focused on screening implementation (Phanguphangu & Ross, 2025) and diagnostic outcomes (Kuschke et al., 2020), with limited longitudinal data describing whether children ultimately receive amplification, medical intervention, habilitation referrals, or sustained audiological follow-up. Furthermore, few studies have characterised management needs among medically complex children or those presenting with conductive and mixed hearing losses commonly observed in tertiary paediatric settings (Van Zyl et al., 2022).

A preceding study at the Red Cross War Memorial Children’s Hospital (RCWMCH) described delayed screening and diagnostic timelines relative to international benchmarks, a predominance of infectious and neurological risk factors, and a high burden of conductive and mild-to-moderate hearing losses (Kuschke et al., 2026). These characteristics suggest that management pathways in this population may differ substantially from those described in high-income EHDI settings, where permanent bilateral sensorineural hearing loss predominates. These findings also raise important questions regarding subsequent management: Do children have access to amplification? Are multidisciplinary referrals enacted? Is follow-up sustained?

Timely identification and intervention have been associated with improved speech, language, vocabulary, and broader developmental outcomes in children with hearing loss (Ching et al., 2019; JCIH, 2019; Yoshinaga-Itano et al., 2017). However, paediatric hearing loss management in lower-resourced contexts must account for conductive pathology requiring medical intervention, as well as Deaf or Hard of Hearing Plus (DHH+) presentations, where hearing loss co-occurs with additional developmental, neurological, cognitive, or physical disabilities. These medically and developmentally complex presentations may require coordinated multidisciplinary management and may influence diagnostic, amplification, and intervention pathways.

This study, therefore, aimed to describe management pathways and outcomes of children diagnosed with hearing loss following NIHS at RCWMCH, with the following specific objectives:

- To characterise types and degrees of hearing loss requiring management.
- To describe amplification fitting rates and types.
- To identify referral patterns to medical and early intervention services.
- To describe follow-up attendance and audiological management needs.

By examining amplification uptake, referral patterns, and continuity of follow-up within a tertiary South African paediatric cohort, this study aims to contribute contextually relevant evidence regarding the strengths and limitations of post-diagnostic hearing healthcare delivery in a lower-resourced setting.

## Research methods and design

### Study design

A retrospective descriptive cohort study was conducted between August 2019 and August 2024.

### Study setting

The study was undertaken at RCWMCH, a tertiary paediatric referral hospital in the Western Cape, South Africa, providing diagnostic audiology, amplification services, and multidisciplinary referrals. The hospital serves a predominantly public-sector population. RCWMCH serves a large catchment area within the Western Cape Province, and receives referrals from various sources, including maternity and obstetric units, schools, multidisciplinary teams, ear-, nose-, and throat (ENT) specialists, and physicians. A representative sample of the Western Cape could thus be obtained.

### Study population

Clinical records of children under 6 years were included:

- Had documented the NIHS results.
- Underwent diagnostic assessment.
- Were diagnosed with hearing loss.
- Received audiological management and/or follow-up.

Only children with documented NIHS results in the Audiology Department’s electronic database or clinical records were eligible for inclusion. The study, therefore, reflects a retrospective cohort of available documented NIHS cases within the tertiary audiology service, rather than all children seen by the department during the study period. Of the children with documented NIHS results, all children who underwent subsequent diagnostic audiological assessment and met the inclusion criteria during the study period were included.

### Sampling strategy

A retrospective review of all available clinical records meeting the inclusion criteria was conducted using the RCWMCH database and clinical records.

### Data collection

Data were extracted from the RCWMCH electronic database, which routinely captures demographic and clinical data of patients as they are seen, and from the clinical records of included patients. A self-developed Microsoft Excel version 16 spreadsheet was used to collate and clean the data for analysis with IBM Statistical Package for the Social Sciences (SPSS) for Windows, Version 31.0 (IBM Corporation, Armonk, New York, United States [US]). Variables included demographic characteristics, risk factors, diagnostic outcomes, amplification fitting, referral pathways, and follow-up attendance.

Referral patterns referred to documented referrals made by the audiology team to additional healthcare or intervention services, including otolaryngology, speech-language therapy, genetics, cochlear implantation programmes, and early intervention services. Referrals reflected the referral recommendations or referral letters documented within the clinical record and did not necessarily indicate confirmed attendance at the referral site.

Follow-up attendance was defined as documented return attendance for scheduled audiological reassessment, monitoring, intervention, or management appointments within the Audiology Department following initial diagnostic evaluation.

### Data management and reliability

Data were captured using anonymised unique patient identifiers. Data were entered and maintained by four audiologists working in the Audiology Department, using departmental data-capturing protocols. All four audiologists were trained by the Head of the Audiology Department to ensure that data were captured uniformly. To enhance data reliability, extracted data were reviewed for completeness and consistency, and random cross-checks against original clinical records were conducted by the primary investigator to minimise transcription and extraction errors. A secure password-protected database with routine backups was used.

### Data analysis

Descriptive statistics were calculated using IBM SPSS Statistics (version 31). Continuous variables were summarised using means and standard deviations. Categorical variables were reported as frequencies and percentages.

### Ethical considerations

Ethical clearance to conduct this study was obtained from the University of Cape Town Faculty of Health Sciences Human Research Ethics Committee (No. HREC441/2022). This was a retrospective record review study. As the study involved retrospective analysis of routinely collected anonymised clinical data, a waiver of informed consent was granted by the ethics committee in accordance with institutional and hospital research governance procedures. No personally identifiable patient information was included in the dataset used for analysis.

## Results

### Patient population characteristics

**FIGURE 1:**
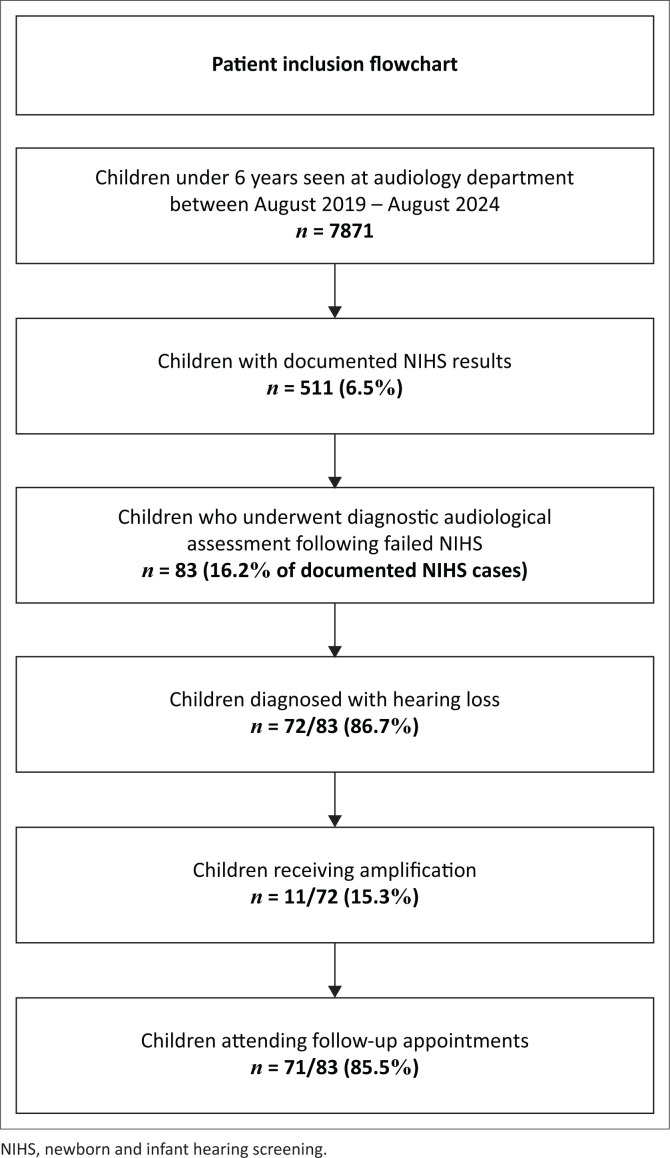
Patient inclusion flowchart.

The total number of patients under 6 years who were seen at RCWMCH Audiology during August 2019 – August 2024 was 7871. Of those, only 511 (6.5%) had documented NIHS results. The relatively low proportion of children with documented NIHS results likely reflects a combination of inconsistent screening implementation across referral settings, variable documentation practices, and incomplete transfer of screening information into the audiology record system, rather than true screening prevalence within the broader paediatric population. The number of children who went on to receive diagnostic hearing evaluations after failing hearing screening was 83 out of 511 (16.2%) and were included in this study sample. The final sample of 83 children, therefore, represented the entire available cohort of children with documented failed NIHS results who underwent diagnostic evaluation within the department during the review period. Of the 83 children included, 72 (86.7%) were diagnosed with hearing loss following diagnostic evaluation. The mean age at hearing loss diagnosis was 17.3 months (18.4 standard deviation [s.d.]; range 1–56). Figure 1 summarises the study cohort flow and inclusion process.

### Risk factors for hearing loss

Table 1 shows the risk factors for hearing loss of the current study sample (*n* = 83). The infectious (32.5%) and neurological (28.9%) categories accounted for the majority of risk factors in the study sample.

**TABLE 1:** Risk factors associated with hearing loss (*n* = 83).

Risk factor	*n*	*%* †
Infectious	27	32.5
Neurological	24	28.9
Unspecified	17	20.5
Syndromic or genetic	11	13.3
Not recorded	4	4.8

Note: Infectious – Meningitis; rubella; cytomegalovirus; syphilis. Neurological – Hypoxic ischaemic encephalopathy; cerebral palsy; prematurity. Syndromic or Genetic – Down syndrome; Waardenburg syndrome; Apert syndrome; Goldenhar syndrome; Noonan syndrome.

† , *Percentages may not total 100% due to rounding*.

### Diagnostic outcomes

Diagnostic hearing results for the sample are presented in Table 2 in terms of type and degree of hearing loss per ear. Conductive hearing loss accounted for approximately one-quarter of cases per ear. However, a substantial proportion of cases were classified as ‘undetermined’ by hearing loss type (38.6% right ear; 33.7% left ear), reflecting considerable diagnostic uncertainty within this medically complex paediatric cohort. Mild (31.3%) and moderate (33.7%) degrees of hearing loss were the most prevalent severity classifications.

**TABLE 2:** Diagnostic results per ear (*n* = 83).

Variable	Right ear		Left ear	
	*n*	%	*n*	%
**Type of hearing loss**				
Normal	11	13.3	9	10.8
Conductive hearing loss	21	25.3	25	30.1
Sensorineural hearing loss	15	18.1	16	19.3
Mixed hearing loss	1	1.2	1	1.2
Auditory Neuropathy Spectrum Disorder	3	3.6	4	4.8
Undetermined	32	38.6	28	33.7
**Degree of hearing loss**				
Normal	11	13.3	9	10.8
Mild	26	31.3	22	26.5
Moderate	28	33.7	31	37.3
Severe	2	2.4	2	2.4
Profound	8	9.6	9	10.8
Undetermined	8	9.6	10	12.0

### Amplification management

The total number of children with any type or degree of hearing loss after undergoing diagnostic testing was 72 out of 83 (86.7%), and the total number who were subsequently fitted with initial amplification was 11 out of 72 (15.3%). Table 3 shows the different types of initial amplification management for hearing loss in this sample. Most children were fitted with bilateral behind-the-ear hearing aids (72.7%).

**TABLE 3:** Amplification type (*n* = 11).

Laterality and type of amplification	*n*	*%*
Bilateral	8	72.7
Unilateral	3	27.3
BTE	8	72.7
FM	1	9.1
BCHD	2	18.2

BTE, behind-the-ear; FM, frequency modulation system; BCHD, bone conduction hearing device.

### Referrals

The proportion of children requiring onward referral was 65 out of 83 (78.3%). Children could receive more than one referral. Table 4 summarises referral categories as proportions of the 65 children referred, rather than mutually exclusive referral events.

**TABLE 4:** Referrals (*n* = 65).

Referral source†	*n*	%
Ear-nose-throat specialist	53	81.5
Cochlear implantation	6	9.2
Hi-Hopes (home-based early intervention)	5	7.7
Speech-language pathologist	11	16.9
Neuro-developmental clinic	4	6.2
Schools for hearing loss	4	6.2

† , Children could receive multiple referrals; percentages therefore exceed 100%.

### Follow-up sessions

A total of 71 out of 83 (85.5%) children attended follow-up appointments after diagnostic testing. Table 5 details the type of follow-up sessions that patients attended. The mean number of follow-up visits over the study period was 4.6 (s.d.: 4.5; range 1–24). Given the skewed distribution of follow-up visits, the median number of follow-up appointments was 3 (interquartile range: 2–5).

**TABLE 5:** Follow-up sessions (*n* = 71).

Type of follow-up session	*n*	%
Earmold impressions	12	16.9
Retesting	69	97.2
Hearing aid repairs	3	4.2
Amplification adjustments	4	5.6
Real-ear measurements	10	14.1
Retubing	4	5.6

## Discussion

This study describes post-diagnostic referral pathways and management outcomes following NIHS within a South African public-sector tertiary hospital context, highlighting the continuum from diagnosis to intervention in a medically and socially complex paediatric population. Although follow-up attendance was relatively high, important gaps emerged in amplification uptake and referral to structured early intervention services, pointing to ongoing challenges in translating early detection into timely (re)habilitative action.

### Risk profile and contextual burden

Neurological and infectious risk factors accounted for approximately two-thirds of the sample. Conditions such as hypoxic-ischaemic encephalopathy, prematurity, meningitis, and congenital infections remain disproportionately prevalent in South Africa compared to high-income settings (Loucaides et al., 2025). Recent South African literature continues to emphasise the contributions of perinatal complications, neonatal intensive care admissions, and the infectious disease burden to paediatric hearing loss profiles (Khoza-Shangase, 2019; Swanepoel & Clark, 2019). These findings reflect broader systemic challenges in maternal and neonatal health and highlight the intersection between public health burden and paediatric hearing loss.

Thirteen per cent of children presented with syndromic or genetic conditions, consistent with the DHH+ population described in international literature (Cupples et al., 2018). The high proportion of neurological comorbidity aligns with emerging descriptions of complex DHH+ presentations within South African public health services; however, evidence specific to local service-delivery contexts remains limited, and further South African-focused research is needed to substantiate this comparison. Management in such cases requires coordinated multidisciplinary care, as hearing loss often co-exists with cognitive, motor, or developmental impairments.

### Conductive pathology and ear, nose, and, throat integration

Approximately one-quarter of the sample presented with conductive hearing loss. High rates of middle ear disease in South African children have been widely reported, particularly those from lower socioeconomic communities (Biagio et al., 2014; Khoza-Shangase & Munyembate, 2025; Sebothoma & Khoza-Shangase, 2022). Environmental overcrowding, recurrent upper respiratory infections, and limited primary healthcare follow-up contribute to this burden. The high rate of otolaryngology referral (81.5%) reflects appropriate interprofessional management. One possible explanation for the relatively low amplification rate is that conductive hearing loss may have resolved following medical or surgical treatment; however, this cannot be confirmed in the present dataset because ENT treatment outcomes were not systematically captured. Fluctuating conductive pathology may nevertheless place children at risk of inconsistent auditory access during critical periods of language acquisition. Strengthened audiology–ENT integration and streamlined follow-up pathways are therefore essential.

### Undetermined hearing loss type

A notable proportion of cases were classified as undetermined by type. While this likely reflects the clinical realities of testing medically fragile infants and toddlers, including incomplete electrophysiological data, inconsistent behavioural responses, developmental delay, and fluctuating middle ear status, procedural and contextual factors must also be considered. South African literature has highlighted variability in paediatric audiological assessment practices, challenges in obtaining complete diagnostic test batteries, and resource limitations affecting diagnostic certainty in young children (Sebothoma & Khoza-Shangase, 2022; Störbeck et al., 2023). Limited test time, poor follow-up attendance, equipment availability, and clinician variability may all contribute to incomplete classification.

Importantly, only a small proportion of cases were undetermined by degree, suggesting that management decisions could still often be guided by estimated severity despite incomplete diagnostic specificity. These findings highlight the importance of pragmatic and flexible clinical decision-making in lower-resourced contexts, where delaying intervention until complete diagnostic certainty is achieved may risk delaying critical auditory access during early developmental periods.

### Amplification uptake

Only 15.3% of children diagnosed with hearing loss received amplification. This figure must be interpreted within context. A significant proportion of losses were conductive and may have resolved following ENT intervention. Nearly 10% of ears demonstrated profound hearing loss, and delayed age of diagnosis (mean 17 months) likely reduced alignment with international EHDI benchmarks (JCIH, 2019). Late diagnosis has been consistently reported in the South African NIHS literature (Khoza-Shangase, 2019; Kuschke et al., 2020), thereby limiting the timely initiation of amplification.

Structural and economic constraints cannot be overlooked. Hearing aids, bone conduction hearing devices, and FM systems are procured through state tender processes and are costly, with limited annual allocations. Bone conduction hearing devices, in particular, are resource-intensive and often restricted in availability. Additionally, funding responsibilities for assistive listening devices frequently intersect between the Departments of Health and Education, potentially delaying provision.

Socioeconomic barriers, transport costs, caregiver employment constraints, and family communication preferences have been suggested as potential influences on amplification uptake (Cupples et al., 2018). However, the present study did not collect data on communication modality or family language choices; therefore, these factors should be interpreted only as contextual considerations, supported by the broader literature rather than by findings from this dataset. The relatively low amplification uptake observed in this cohort is consistent with reports from other lower-resourced settings, where delays in device provision, limited funding pathways, and fragmented early intervention systems contribute to reduced hearing aid fitting rates (McPherson, 2012; Olusanya et al., 2024). However, these findings may also reflect broader service-delivery challenges, including delayed referral pathways, repeated diagnostic reassessment, and the complexity of decision-making in medically fragile children (Olusanya et al., 2024). Collectively, these factors suggest that improving amplification uptake requires both resource investment and strengthened coordination across the diagnostic-to-intervention continuum.

### Follow-up and continuity of care

Although follow-up attendance rates were comparatively high relative to reports from some South African public-sector screening programmes (Phanguphangu & Ross, 2025), most visits involved ongoing reassessment rather than habilitative intervention, reflecting the complexity and instability of hearing status in this cohort.

### Early intervention and cochlear implantation referrals

Relatively low referral rates to cochlear implantation and structured early intervention programmes warrant attention. Access to cochlear implantation services in South Africa remains centralised, with geographic inequities affecting rural families (Khoza-Shangase & Munyembate, 2025; Petrocchi-Bartal et al., 2025). Six children were referred for cochlear implantation evaluation. However, interpretation of this figure requires consideration of candidacy criteria. Cochlear implantation is typically indicated for children with bilateral severe-to-profound sensorineural hearing loss who derive limited benefit from amplification. In the present dataset, it is not possible to determine precisely how many of the six referred children met strict audiological candidacy criteria for implantation, as degree-specific subgroup data were not consistently available for all cases. Therefore, it is unclear whether this referral rate represents under-referral, appropriate triage based on hearing severity, or constraints related to service accessibility and eligibility pathways.

Recent South African data call for decentralised and community-based early hearing intervention models (Kuschke et al., 2021; Swanepoel & Clark, 2019) that highlight the importance of strengthening referral pathways beyond tertiary hospital walls. Without robust integration of early intervention, the developmental gains associated with early identification may not be realised.

### Limitations

The relatively low proportion of children with documented NIHS results within the departmental database limits the generalisability of the findings and likely reflects variability in screening implementation and documentation practices across referral settings. It is important to highlight that this study represents a retrospective cohort of documented NIHS cases available within a tertiary audiology service, rather than all children undergoing NIHS within the broader healthcare system.

The relatively high proportion of ‘undetermined’ hearing loss classifications limits interpretation of hearing loss type distributions and likely reflects the complexity of paediatric diagnostic assessment within medically fragile populations and retrospective clinical datasets.

The current study was unable to track outcomes beyond the hospital setting, including whether otolaryngology referrals were attended, whether medical or surgical management of conductive hearing loss was completed, and whether conductive losses resolved over time. This also limited the interpretation of amplification decisions, cochlear implantation referrals, and longer-term intervention outcomes.

### Recommendations for practice and future research

The findings of this study highlight several important implications for paediatric hearing healthcare within South African public-sector contexts. Strengthening integration between audiology, otolaryngology, early intervention, and developmental services may improve continuity of care for medically complex children. Earlier and more standardised referral pathways for amplification and habilitative services should be prioritised, particularly for children at risk of delayed auditory access.

Given the high burden of conductive and medically complex hearing loss observed, contextually appropriate management protocols that accommodate fluctuating hearing status and developmental comorbidities are needed. Expanding community-based and decentralised early intervention services may further reduce geographic and socioeconomic barriers to care.

Future research should include prospective longitudinal studies evaluating developmental, communication, and educational outcomes following diagnosis and intervention. Further investigation into barriers to amplification uptake, reasons for loss to follow-up, and variability in paediatric audiological assessment practices within South African public healthcare settings would also be valuable. Future studies should explicitly stratify referrals by degree and configuration of hearing loss to enable clearer interpretation of cochlear implant service uptake.

## Conclusion

This study sheds light on important gaps along the continuum of paediatric hearing healthcare following NIHS within a South African tertiary hospital context. Clinically, the findings of this study emphasise the need for strengthened integration between audiology, otolaryngology, and early intervention services to ensure continuity of care, particularly for children with fluctuating or medically complex hearing loss. Improved coordination across services may help reduce delays in intervention and support more consistent auditory access during critical periods of speech and language development.

More broadly, the study highlights the influence of systemic constraints, including resource limitations and fragmented care pathways, on paediatric hearing healthcare delivery in lower-resourced settings. Addressing these barriers will require improved interprofessional collaboration, enhanced service planning, and allocation of assistive hearing technologies.

Future research should prioritise prospective, longitudinal designs to better track outcomes following diagnosis, including hearing stability, amplification use, adherence to ENT recommendations, and developmental outcomes. In addition, studies examining caregiver decision-making, service access barriers, and the real-world implementation of early intervention pathways would further inform improvements in NIHS programmes.
